# RETRACTED: Nabwey et al. A Comprehensive Review of Nanofluid Heat Transfer in Porous Media. *Nanomaterials* 2023, *13*, 937

**DOI:** 10.3390/nano16160986

**Published:** 2026-08-10

**Authors:** Hossam A. Nabwey, Taher Armaghani, Behzad Azizimehr, Ahmed M. Rashad, Ali J. Chamkha

**Affiliations:** 1 Department of Mathematics, College of Science and Humanities in Al-Kharj, Prince Sattam Bin Abdulaziz University, Al-Kharj 11942, Saudi Arabia; 2 Department of Basic Engineering Science, Faculty of Engineering, Menoufia University, Shebin El-Kom 32511, Egypt; 3 Department of Engineering, West Tehran Branch, Islamic Azad University, Tehran 1477893855, Iran; armaghani.taher@yahoo.com (T.A.); behzadazizimehr@wtiau.ac.ir (B.A.); 4 Department of Mathematics, Faculty of Science, Aswan University, Aswan 81528, Egypt; am_rashad@yahoo.com; 5 Faculty of Engineering, Kuwait College of Science and Technology, Doha District, Kuwait City 35004, Kuwait; a.chamkha@kcst.edu.kw

The journal retracts the article, “A Comprehensive Review of Nanofluid Heat Transfer in Porous Media” [1] cited above.

Following publication, concerns were brought to the attention of the publisher regarding the reliability of statements, the relevancy of a number of cited references, and permissions to use certain data representations contained within this review article [1].

In accordance with the journal’s standard procedures, the Editorial Office and Editorial Board conducted an investigation into this review article. The investigation confirmed that a substantial proportion of the cited references could not be considered appropriate or valid in the context of the review. Additional concerns regarding the accuracy of the content and potential copyright issues were identified and could not be fully resolved. Although the authors cooperated with the investigation, the explanations and supporting materials provided were not deemed sufficient by the Editorial Board to resolve the identified concerns. As a consequence, the Editorial Board has lost confidence in the reliability of the reported findings and has decided to retract this publication in accordance with MDPI’s retraction policy.

This retraction was approved by the Editor-in-Chief of the *Nanomaterials* journal.

The authors have been informed of this retraction and have indicated their disagreement with this decision.
